# Antidepressant efficacy is correlated with plasma levels: mega-analysis and further evidence

**DOI:** 10.1097/YIC.0000000000000386

**Published:** 2021-12-13

**Authors:** Lorenzo Cellini, Domenico De Donatis, Gerald Zernig, Diana De Ronchi, Giancarlo Giupponi, Alessandro Serretti, Hart Xenia, Andreas Conca, Vincenzo Florio

**Affiliations:** a Department of Biomedical and Neuromotor Sciences, University of Bologna, Bologna; b Department of Psychiatry, Comprensorio Sanitario di Bolzano, Bolzano, Italy; c Department of Psychiatry 1, Medical University of Innsbruck, Innsbruck, Austria; d Department of Molecular Neuroimaging, Central Institute of Mental Health, University of Heidelberg, Mannheim, Germany

**Keywords:** antidepressant response, major depression, mirtazapine, selective serotonin reuptake inhibitor, serotonin-norepinephrine reuptake inhibitor, serum concentration, therapeutic drug monitoring

## Abstract

The debate around optimal target dose for first-line antidepressants (ADs) is still ongoing. Along this line, therapeutic drug monitoring (TDM) represents one of the most promising tools to improve clinical outcome. Nevertheless, a few data exist regarding the concentration-effect relationship of first-line ADs which limits TDM implementation in routine clinical practice. We conducted the first patient-level concentration-response mega-analysis including data acquired by us previously and explored the concentration dependency of first-line AD (206 subjects). Further, new data on mirtazapine are reported (18 subjects). Hamilton Depression Rating Scale-21 administered at baseline, at month 1 and month 3 was used as the measure of efficacy to assess antidepressant response (AR). When pooling all four first-line ADs together, normalized plasma levels and AR significantly fit a bell-shaped quadratic function with a progressive increase of AR up to around the upper normalized limit of the therapeutic reference range with a decrease of AR at higher serum levels. Our results complement the available evidence on the issue and the recent insights gained from dose-response studies. A concentration-dependent clinical efficacy, such as previously demonstrated for tricyclic compounds, also emerge for first-line ADs. Our study supports a role for TDM as a tool to optimize AD treatment to obtain maximum benefit.

## Introduction

First-line antidepressants (ADs), such as selective serotonin reuptake inhibitors (SSRIs), serotonin-norepinephrine reuptake inhibitors (SNRIs) and noradrenergic and specific serotonergic antidepressant (NaSSA), are extensively prescribed worldwide in clinical practice and represent the first pharmacological option in major depressive disorder (MDD). Despite their unquestioned efficacy, dose-response correlational studies repeatedly failed to identify a dose effect until recent years (Hieronymus *et al.*, 2016). On the contrary, physicians routinely continue AD titration above the minimum licensed dose before switching to another AD, which would be an irrational strategy in the presence of a flat dose-response relationship. Clinical guidelines reflect this uncertainty regarding the optimal dose of AD, as some, such as the American Psychiatric Association (APA) Guidelines, suggest titration up to the maximum tolerated dose, whereas others highlight the absence of an established dose-response relationship, such as the one provided by the UK National Institute of Health and Care Excellence (Gelenberg *et al.*, 2010; National Collaborating Centre for Mental Health (UK), 2010). However, more recent meta-analyses have suggested a dose effect for efficacy, tolerability and acceptability, and suggest to incorporate these pieces of evidence and update current practice guidelines (Hieronymus *et al.*, 2016; Furukawa *et al.*, 2019).

Evidence gained from dose-response studies may carry biases because it is not possible to control the variability of plasma levels induced by pharmacokinetic parameters. As a result, there is a growing interest in therapeutic drug monitoring (TDM), with mixed initial results when considering AD pharmacotherapy specifically. TDM is supposed to play a role as an aid to clinicians in defining an appropriate AD prescription, based on the assumption that clinical efficacy correlates better to drug plasma levels than doses (Ostad Haji *et al.*, 2012; Fiaturi and Greenblatt, 2019). However, AD concentration-response studies have received little attention given that previous registration trials were not always able to detect a correlation between AR and the plasma levels due to the high noise-to-signal ratio as clarified in a 2014 seminal position article (Preskorn, 2014). The more recent Consensus Guidelines for Therapeutic Drug Monitoring in Neuropsychopharmacology suggest the application of TDM when there is an established therapeutic range, an increased risk of intolerance or intoxication or a known concentration-effect relationship of the prescribed drug (Hiemke *et al.*, 2018). Most tricyclic compounds (TCAs), for example, meet all these fundamental conditions, also considering that a curvilinear concentration-response relationship has been widely demonstrated (Perry *et al.*, 1987; Preskorn and Fast, 1991; Perry *et al.*, 1994; Müller *et al.*, 2003; Hiemke *et al.*, 2018).

On the contrary, for first-line ADs, despite specific therapeutic ranges having been established, there is no evidence to support routine TDM, with the only exception of citalopram (Ostad Haji et al., 2011, 2013; Hiemke *et al.*, 2018). Moreover, for some of the first-line ADs (e.g. mirtazapine) no concentration-effect relationship has been demonstrated yet (Hiemke *et al.*, 2018; Fiaturi and Greenblatt, 2019), or just a few naturalistic studies exist (Charlier *et al.*, 2002; Daviss *et al.*, 2006; Ostad Haji et al., 2011, 2013; Florio *et al.*, 2017; De Donatis et al., 2019, 2021). Therefore, a step toward a better definition of the underlying correlation of AD response on plasma levels is needed to efficiently guide treatment in clinical practice. Mirtazapine is a case in point, being at standard doses (15-45 mg/die) one of the most effective AD in head-to-head studies, with a likely faster onset of action (Watanabe *et al.*, 2011; Wang *et al.*, 2018; Cheng *et al.*, 2020). Recent findings suggest that the dose-efficacy curve increases up to a dose of 30 mg/die which then decreases showing optimal acceptability in the lower range of their licensed dose (Furukawa *et al.*, 2019). Unfortunately, there is a scarcity of both TDM studies and PET in the living human brain (Smith et al., 2007, 2009; Finnema *et al.*, 2015). TDM guidelines state that no definitive evidence relates mirtazapine serum concentration (30–80 ng/ml) to clinical outcome (Hiemke *et al.*, 2018). At state of the art, no concentration-effect relationship between serum levels and the therapeutic outcome has been proven. Moreover, no clear-cut correlation with the in vivo occupation of the specific target receptor has yet been shown. (Schoretsanitis *et al.*, 2018). Consistently, in the latest TDM guidelines, mirtazapine only reached a ‘Level 2’ of recommendation given the need for further investigation on these issues (Hiemke *et al.*, 2018).

To fill this gap, we aimed at examining plasma levels of first-line ADs. To fully cover all major first-line AD classes, we first present our preliminary results exploring the association between a NaSSA (mirtazapine) and antidepressant response (AR) in a small sample of MDD outpatients in a naturalistic setting. Then, we conducted a posthoc patient-level mega-analysis comprising all our previous works concerning three different AD classes (SSRI, SNRI and NaSSA) investigating the concentration-response relationship of first-line AD.

## Methods

### Clinical sample and experimental measures

Subjects were selected from outpatients who met DSM-5 criteria for MDD in the Psychiatric Unit of Bolzano during the period 2018-2019. Inclusion criteria were a diagnosis of current major depressive episode according to DSM V criteria (American Psychiatric Association, 2013), with a Hamilton Depression Rating Scale-21 items (HAMD-21) score greater than or equal to 14 (Hamilton, 1967). Exclusion criteria were (a) substance dependence with drug consumption during the last 3 months, (b) pregnancy, (c) suicidality, (d) unstable medical conditions and (e) age less than 18 years. Patients were given treated with mirtazapine monotherapy (benzodiazepine use was allowed at a dosage lower than diazepam at 10 mg equivalent) in a naturalistic setting. We then performed a 3-month follow-up and clinical assessment using HAMD-21. Plasma concentrations were measured by liquid chromatography tandem mass spectrometry at the University of Innsbruck (Saint-Marcoux *et al.*, 2007). The study was approved by the local ethical committee (28/2004) and the informed consent was signed by all the subjects.

We then conducted a mega-analysis pooling individual-level data from all our previous studies. Mega-analyses in fact, when feasible, combining raw data into a larger, single sample allow retention of more detailed information and avoid several within-study assumptions than other designs (i.e. meta-analyses) (Boedhoe *et al.*, 2019; Eisenhauer, 2021). Moreover, it enables to eliminate methodological heterogeneity between the incorporated studies enhancing statistical power (Stewart and Tierney, 2002; Riley *et al.*, 2007; Shrier *et al.*, 2007). To maintain homogeneity, we included data from our previous publication which are homogeneous in terms of patient population, clinical setting and prescription style (Florio *et al.*, 2017; De Donatis et al., 2019, 2021). All plasma levels included in the compilation were measured only one time per patient at trough concentration and under steady-state condition.

Because in literature there is no validated formula for equivalence between serum concentration of different ADs, we normalized plasma levels to an ideal therapeutic reference range (0-100 ng/ml), where 0 ng/ml represents the lower limit of the ideal therapeutic reference range and 100 the upper one, respectively. Plasma levels of all four examined ADs show linear pharmacokinetics at therapeutic ranges (Hiemke *et al.*, 2018). Negative values represent subtherapeutic plasma levels. Values two-fold higher than the upper limit of the ideal therapeutic reference range (over 200 ng/ml) represent the equivalent of the ‘laboratory alert levels’ above which severe drug adverse reactions or intoxications are to be expected in accordance with the updated consensus guidelines (Hiemke *et al.*, 2018). As it is well established that the achievement of a minimum of enzyme blockage is a mandatory condition to AD effect, and that concentrations above the laboratory alert level are considered as an indicator of potential abnormalities such as drug-drug interactions, genetic polymorphisms, or diseases of organs involved in drug clearance (Hiemke *et al.*, 2018), we also conducted a pooled analysis where only concentrations within the 0-200 ng/ml range are considered.

For further details on associated clinical variables and experimental measures of the samples included in the mega-analysis, refer to Table 3 and to our original works (Florio *et al.*, 2017; De Donatis et al., 2019, 2021).

### Statistical analyses

Linear regression analysis was performed to calculate the association between plasma levels and AR at both 1 and 3 months. We repeated the same analysis within the 0-200 ng/ml range. Finally, nonlinear least-squares regression with the Levenberg-Marquardt algorithm was applied to find the best fitting model explaining the association between plasma level and AR. Further, we repeat the analysis according to and accounting and stratifying for known interaction factors such as age, sex and ethnicity (Hiemke *et al.*, 2018). Data analyses were performed using SPSS Statistics for Windows, version 20.0. (SPSS Inc., Chicago, Illinois, USA). All *P* values were two tailed and statistical significance was conservatively set at *P* < 0.05.

## Results

Sociodemographic and clinical features of the sample are reported in Table 1. In the total sample, we did not find an association between mirtazapine plasma levels and AR response both at month 1 (*P* = 0.27, *r* = 0.27) and at month 3 (*P* = 0.01, *r* = 0.59). We repeat the analysis excluding two patients who showed extremely low plasma levels less than or equal to 8 ng/ml, likely for lack of compliance (*P* = 0.06, *r* = 0.48 and *P* = 0.006, *r* = 0.66, respectively). Despite the lack of statistical significance throughout the entire follow-up period due to the lack of statistical power, the preliminary results show a promising pattern especially considering the small sample involved (Table 1) and in line with those previously reported for escitalopram, duloxetine and venlafaxine. We argue that further considerations, specifically concerning mirtazapine concentration, could be drawn when more data can be acquired.

**Table 1:** Clinical features of the mirtazapine sample

Clinical features (*n* = 18)	
Age (years)	58.11 ± 16.85
Sex	
Males	38.9%
Females	61.1%
Ethnicity	
Caucasian	100%
SCM (ng/ml)^a^	36.81 ± 20.28^b^
HAMD-21 baseline	20.83 ± 5.22
HAMD-21 month 1	15.76 ± 4.79
HAMD-21 month 3	10.39 ± 4.05
Patients with >20% improvement at month 1 (%)	13 (72%)
Patients with >50% improvement at month 3 (%)	9 (50%)

HAMD-21, Hamilton Depression Rating Scale-21.

a Two patients showed extremely low or not quantifiable SCM (likely for lack of compliance); thus, they were excluded from the analysis.

b SCM, serum concentration of mirtazapine.

### Mega-analysis results

Clinical features and more common variables of the global sample are reported in Table 2 as well as individually for each AD (Tables 1 and 3). Preliminary analysis reveals no difference in terms of sex distribution, whereas a slight difference was observed for age distribution between escitalopram and the other samples (Table 3). Likewise, a small difference was observed for baseline severity; venlafaxine sample shows slightly higher HAMD-21 scores [95% confidence interval (CI): 25.54-27.07] compared to all other ADs (95% CI: 23.12-24.5) and duloxetine sample a higher baseline severity with respect to the mirtazapine one, although NS. After pooling all raw data together, no correlation was found between AR and age, sex, or other sociodemographic and clinical features.

**Table 2:** Clinical features of the global sample

Clinical features (*n* = 206)	
Age (years)	58.11 ± 16.85
Sex	
Males	40.8%
Females	59.2%
Ethnicity	
Caucasian	94.2%
Others	5.8%
Normalized plasma levels (ng/ml)	45.42 ± 42.32
Concomitant benzodiazepine treatment	
Yes	78.2%
No	21.8%
HAMD-21 baseline	24.44 ± 4.12
HAMD-21 month 1	18.88 ± 4.60
HAMD-21 month 3	14.96 ± 5.00
Patients with >20% improvement at month 1 (%)	119 (57.8%)
Patients with >50% improvement at month 3 (%)	67 (32.5%)

HAMD-21, Hamilton Depression Rating Scale-21.

**Table 3:** Clinical features divided by antidepressant samples

	Escitalopram (*n* = 70)	Duloxetine (*n* = 66)	Venlafaxine (*n* = 52)
Age (years)	46.20 ± 16.63	56.42 ± 14.55	55.73 ± 13.81
Sex			
Males	40%	39%	44%
Females	60%	61%	56%
Ethnicity			
Caucasian	91.4%	95.45%	-
Others	8.6%	4.55%	-
Normalized plasma levels (ng/ml)	30.33 ± 28.36	48.72 ± 45.41	61.18 ± 47.84
Concomitant benzodiazepine treatment			
Yes	22.86%	31.82%	78.2%
No	77.14%	68.18%	21.8%
HAMD-21 baseline	23.80 ± 4.50	24.64 ± 3.48	26.31 ± 2.75
HAMD-21 month 1	17.18 ± 5.03	19.67 ± 3.75	21.29 ± 3.29
HAMD-21 month 3	13.73 ± 5.56	15.91 ± 4.47	16.98 ± 3.60
Patients with >20% improvement at month 1 (%)	66%	53%	48%
Patients with > 50% improvement at month 3 (%)	42%	26%	35%

HAMD-21, Hamilton Depression Rating Scale-21.

Normalized plasma levels show high interindividual variability (mean 32.63 ± 57.45 ng/ml) and were positively correlated with age (*P* = 0.001, *r* = 0.29), but interestingly we found no significant correlation with sex (*P* = 0.46, *r* = −0.05) or ethnicity.

Finally, we found a significant positive correlation between plasma levels and AR both at the end of the follow-up period at month 3 (*P* = 0.001, *r* = 0.3) and at month 1 (*P* = 0.001, *r* = 0.32).

We conducted further exploratory analyses taking into account the recommended concentration therapeutic range (i.e. 0-100 ng/ml) and stratifying for variables with a known potential for interaction in the association between plasma levels and response.

Within the recommended therapeutic range, we still found a positive correlation between plasma levels and AR both at 1 month (*P* = 0.001, *r* = 0.31) and 3 months (*P* = 0.00, *r* = 0.39).

Stratifying per age, we found for ages greater than 65 years a similar positive correlation at 1 month (*P* = 0.001, *r* = 0.46) and at 3 months (*P* = 0.001, *r* = 0.28).

Furthermore, we tested which model best describes the relationship between plasma levels and AR. We found that the quadratic function AR = *a* + (PL − PL2), relative to other possible models, including the logistic one, explained a higher percentage of variance both at month 1 (*P*<0.001, *F* = 16.556, *r* = 0.393) and at month 3 (*P* < 0.001, *F* = 28.47, *r* = 0.455). Repeating the analysis, considering concentrations within the 0-200 ng/ml range, gives similar results (respectively *P* < 0.001, *F* = 12.59 and *P* < 0.001, *F* = 7.9)

## Discussion

The aim of the present study was to investigate a possible drug-response relationship for first-line AD to clarify the usefulness of TDM for AD treatment optimization. It stands out in many regards: it focuses on TDM and concentration-response relationship eliminating the bias due to the high PK interindividual variability of dose-response studies; it is based on pooled patient-level data; homogeneous data were all collected from a real-world and monocentric naturalistic setting showing comparable sample size between drugs with the exception of the mirtazapine (*n* = 18) which is significantly smaller than the others.

**Fig. 1:**
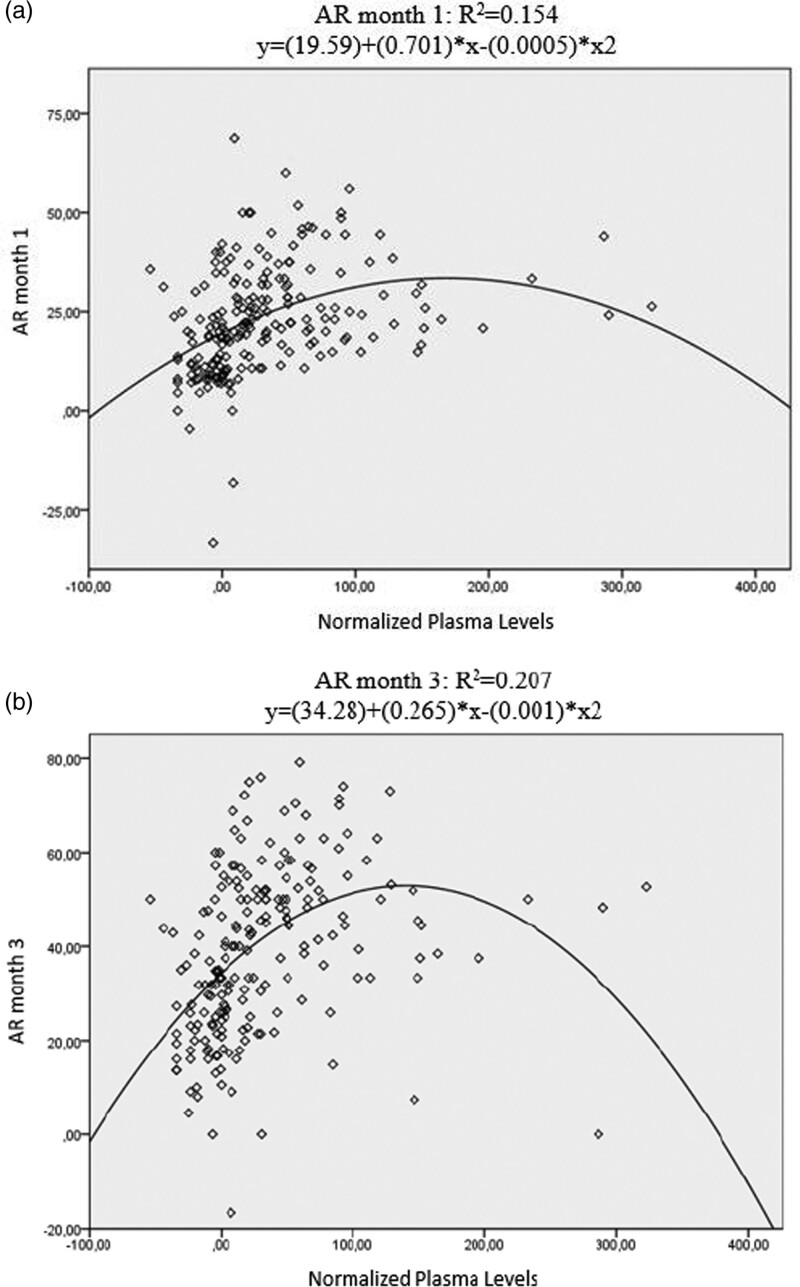
Best fitting model. (a) AR month 1; (b) AR month 3. AR, antidepressant response.

**Fig. 2:**
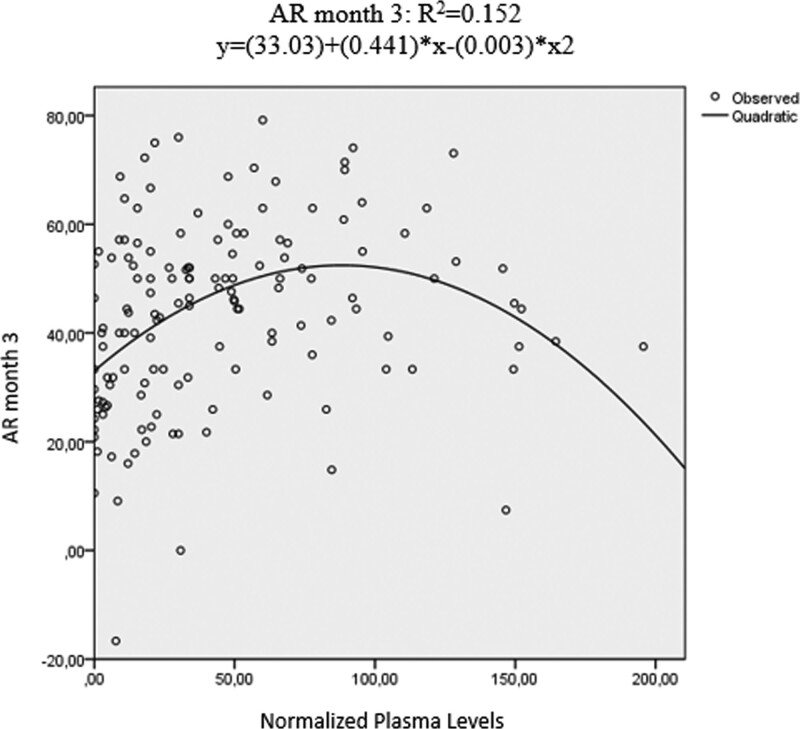
Best fitting model considering concentrations within the 0-200 ng/ml range.

First, the high interindividual variability found in previous works has been replicated after normalization (mean 32.63 ± 57.45 ng/ml). Interestingly, a positive correlation emerges between plasma concentrations and age, highlighting the importance of TDM especially in the elderly, where no reliable strategy to calculate a proper dose exists. A better description of the wide PK interindividual variability caused by the CYP450 system is provided in our previous studies (Florio *et al.*, 2017; De Donatis et al., 2019, 2021). However, we found no correlation between normalized plasma levels and sex. Similarly, data show no correlation between plasma levels and ethnicity despite the fact that both are known factors involved in the relationship between plasma levels and AR (Hiemke *et al.*, 2018); this could be due to the small sample size, especially for ethnicity, since most of our sample is homogenously Caucasian. Finally, we found a direct association between plasma levels and AR both at month 1 and month 3, consistent with our previous preliminary studies (Florio *et al.*, 2017; De Donatis et al., 2019, 2021). The best fitting model follows a bell-shaped quadratic function (Fig. 1). The aforementioned associations have also been confirmed when excluding from the analysis all patients with plasma levels below the lower reference range threshold normalized at 0 ng/ml (fast metabolizers or more likely because of poor compliance) and above 200 ng/ml. Nonetheless, when we investigated the best fitting curve adopting the latter criteria, the curve shifted toward the left indicating a progressive increase within the normalized therapeutic reference range with the maximum peak reached at around 100 ng/ml (Fig. 2). We observe the same phenomenon when stratifying for age (young adults:18-65 years vs. elderly: >65 years) both at month 1 and at month 3. This is consistent with the formal theoretical definition of the upper bound limit provided by the TDM guidelines: in fact, the upper limit thresholds are obtained by calculation of expected dose-related drug concentration at trough levels attained under approved maximum doses and refer to the concentration where maximum efficiency is expected (Hiemke *et al.*, 2018). Our results are in line with the theoretical calculations because a positive concentration-effect is observable up to around the upper normalized limit of the therapeutic reference range (100 ng/ml) with a subsequent descending part beyond that threshold. Nevertheless, a precise assessment of the upper limit of the therapeutic reference ranges is more prone to error in relation to their dependency on the known concentration-response relationship for each specific AD (Eichentopf *et al*., 2021). Preliminary results derived from a systematic review, for example, show that there is no evidence to update the upper limit specifically for escitalopram (Eichentopf *et al*., 2021). So, the authors highlight the need for more studies and a better refinement of the current therapeutic reference range before considering the use of TDM for dose titration (Eichentopf *et al*., 2021).

We confirmed our previous observations on the lack of efficacy for concentrations below the lower reference range threshold (normalized at 0 ng/ml). This has extensively been observed in the literature considering the pharmacodynamic properties of first-line ADs which require a minimum percentage of enzyme/receptor blockage to provide any clinical efficacy (Meyer *et al.*, 2004).

As previously mentioned, previous publications by other groups were more focused on the dose-response outcome, suggesting a dose-dependent relationship for AD (Bollini *et al.*, 1999; Hieronymus *et al.*, 2016; Jakubovski *et al.*, 2016; Furukawa *et al.*, 2019). A clear-cut difference has not only been proved between placebo and low doses, but also between low doses and higher optimal ones, showing that optimal dosing is indeed superior to the minimum efficacy doses recommended by the manufacturers (Furukawa *et al.*, 2019). Thus, the ongoing debate focuses on whether the optimal dose is closer to the maximum dose allowed by the manufacturer or whether it is closer to the lower range allowed. A recent meta-analysis on the issue favors the latter. Authors identify a stepwise increase in dose correlation up to 20–40 mg of fluoxetine equivalents for SSRI with no further increase in efficacy at higher doses but an increase in dropouts due to adverse effects: this results in an overall optimal acceptability of AD treatment toward the lower end of the licensed range (Hieronymus *et al.*, 2016; Furukawa *et al.*, 2019). Similar results have been found for paroxetine up to 20 mg, sertraline up to 100 mg and mirtazapine up to 30 mg (Hieronymus *et al.*, 2016; Furukawa *et al.*, 2019). Dose efficacy of venlafaxine is somehow an exception because it is the only AD that shows an increase in efficacy at higher doses, likely due to its dual action on serotonin and noradrenaline, with the latter component that might become clinically significant only at high doses (Furukawa *et al.*, 2019). Our previous works on venlafaxine pointed out the same results but focus on the concentration-response relationship (De Donatis *et al.*, 2021). Therefore, in the wider debate regarding the guidelines, Furukawa *et al.* (2019) suggest that there is enough body of evidence to update clinical recommendations, applying the abovementioned target doses as the maximum doses recommended.

Our approach that considers the concentration-response instead of doses could shed a different light on the debate. On one hand, in line with the observation obtained from the dose-response studies, our results dismiss the assumption of a flat concentration-response relationship for first-line ADs. When normalizing and pooling our previous findings, a clear and clinically relevant correlation is observed. On the other hand, our results show a positive dose-response relationship along all the normalized therapeutic reference range of 0-100 ng/ml (Fig. 2). Thus, by eliminating the PK variability intrinsic to dose-response studies, we support the validity of the actual TDM reference range showing a higher efficacy around the upper limit of the therapeutic reference range.

In a real-world setting, defining an optimal target dose would be constraining especially in the presence of very high PK interindividual variability. TDM could, thus, be a valuable tool aiding the clinician in discriminating whether it is rational in a specific patient to further titrate the dose aiming at the upper limit of the therapeutic reference range or conversely to switch AD. Depending on the individual drug clearance, from the same starting point, very different therapeutic choices could be possible. These may diverge from a standardized approach based on a mean target dose (40 mg of fluoxetine equivalent). The current APA guidelines (Gelenberg *et al.*, 2010) recommend titration up to the maximum tolerated dose, coherently to our findings: in fact, indirectly incorporate PK considerations because the maximum dose is usually lower than the maximum licensed one and because it could be seen as a proxy of the maximum tolerated concentration.

Our study is not without limitations. First, we have described a quadratic function to explain a higher percentage of variance compared to various standard models. Nevertheless, it is well known that the underlying pharmacological processes (ligand-receptor interactions and signal transduction) would be better described by two logistic concentration-response combined, an ascending one up to the optimal concentration and a descending one likely due to the adverse effects of higher concentrations (Kenakin, 2018; Zernig and Hiemke, 2020). Psychometric tests, such as HAMD-21, may be only partially sensible to the abovementioned effects because it is neither clinically sound nor reasonable to expect a worsening score at least in the core domains, for example, depressed mood, at high but still licensed doses; at higher concentrations, but below the laboratory alert level, clinical experience is in fact that of expecting more and more side effects but also a better or at least a plateau response in the core domains (Fabbri *et al.*, 2018). In addition, even if is it known from a pharmacological perspective that functions other than the logistic one do not correspond to the underlying processes (i.e. change in the serotoninergic response due to the serotonin transporter blockage), the latter does not directly influence the measure of efficacy (i.e. the HAMD-21) but through many layers of complexity. Instead of the total score of the multidimensional HAMD-21, some authors suggest using a single core domain item as a more sensitive measure to detect AD signal, especially when considering tolerability and acceptability at high doses (Hieronymus *et al.*, 2016). Our approximation with a quadratic function retains a part of the fundamental proprieties of the underlying ligand-receptor process responsible for AR while avoiding problems intrinsically related to the measure of efficacy at high concentrations.

Second, as already outlined in our previous works, the outpatient setting did not guarantee medication compliance or the concomitant assumption of other drugs/foods and/or lifestyles that are known to potentially cause significant plasma level fluctuations. A way to minimize the impact of such variables on the results would have been to allow for multiple measurements across the follow-up period, at least in cases of a subsequent drug prescription, to control for potential interactions. Moreover, the lack of complete information on the smoking habit of each patient recruited in the previous studies represents an important limitation taking into account the well-known influence of CYP1A2 in the metabolism of some of the ADs included in the pooled analysis (Fric *et al.*, 2008).

Third, the current upper limit of the therapeutic reference ranges reflects the lack of further therapeutic improvement due to adverse effects, which only indirectly corresponds to a decreased clinical response (Hiemke *et al.*, 2018). Therefore, upper limits are harder to be accurately estimated especially for ADs with a broad therapeutic index, and the comparability between different ADs is not guaranteed to be as accurate as for the lower threshold (Hiemke *et al.*, 2018). This could introduce a small bias after normalization. For example, when looking at the largest naturalistic TDM database available in literature, it seems that the 90th percentile of their sample showed plasma levels widely below the upper limit of the reference ranges for escitalopram and venlafaxine (Reis *et al.*, 2009); the opposite happens for mirtazapine which could suggest a possible misestimation of the real upper threshold (Reis *et al.*, 2009).

Lastly, because we do not observe a significant correlation between age, sex, ethnicity and AR in our sample, probably because of the relatively small sample size, no multiple regression has been therefore performed. Nonetheless, as they are known interaction factors in explaining the relationship between plasma levels and AR, their effects should not be discarded (Hiemke *et al.*, 2018). For this reason, we performed a separate explanatory analysis stratifying for age where possible, with the disadvantage of decreasing the sample size. However, the sample still remains suitable for the kind of analysis performed. However, our results need to be replicated in other populations. Further, because our analyses were all hypothesis-driven, we decided not to apply any statistical correction because the risk of false-positive findings could not be ruled out.

In conclusion, our mega-analysis showed a clear-cut concentration-response relationship up to the upper limit of the therapeutic reference range. A known concentration-effect relationship has been previously demonstrated for TCA but not for first-line AD, which has limited the implementation of TDM in clinical practice. The numerous reasons for failing to demonstrate concentration-response relationships, a notorious shortcoming of clinical trials in psychiatry, have been discussed in detail elsewhere along with suggestions to avoid the respective pitfalls (Zernig and Hiemke, 2020) There is enough body of evidence to indicate that relying on optimal target doses only may lead to poor clinical decision-making in many of the situations if suggestions derived from oral dose-response studies were to be directly incorporated in the guidelines. Our suggested alternative approach would be that of relying on the maximum tolerated concentration. TDM, being a cost-effective tool, may thus represent the missing link between these two therapeutic strategies according to the principles of precision medicine in psychiatry.

## Acknowledgements

### Conflicts of interest

There are no conflicts of interest.
